# The relationship between women’s empowerment and household food and nutrition security in Pakistan

**DOI:** 10.1371/journal.pone.0275713

**Published:** 2022-10-20

**Authors:** Sidra Ishfaq, Abedullah Anjum, Shahzad Kouser, Glenna Nightingale, Ruth Jepson

**Affiliations:** 1 Scottish Collaboration for Public Health Research and Policy, School of Health in Social Science, University of Edinburgh, Edinburgh, Scotland, United Kingdom; 2 Pakistan Institute of Development Economics (PIDE), Islamabad, Pakistan; 3 Department of Economics, COMSATS University Islamabad, Islamabad, Pakistan; Cornell University, UNITED STATES

## Abstract

Women’s empowerment is considered to play a crucial role in food and nutrition security. We aimed to explore the relationship between women’s empowerment and food and nutrition security, in rural Pakistan. Methods: To estimate women’s empowerment, we developed a Rural Women Composite Empowerment Index incorporating nine domains. For indicators of food and nutritional data we used data of 1879 rural households from Pakistan Rural Household Panel Survey (PRHPS). Food insecurity was measured through a caloric intake approach; nutrition insecurity was measured through recommended nutrient intake (RNI). Using the Rural Women’s Composite Empowerment Index (RWCEI), we employed multi-level mixed-effect regression analysis. Results: The domains of traveling safely (21%), time allocated to tasks (20%), and (lack of) domestic violence (19%) were the most significant domains in defining empowerment of rural woman. The prevalence of food and nutrition insecurity were 33% and 50% respectively. Regression analysis found a positive and significant relationship between women’s empowerment and food and nutrition security–the proportion of household who were food and nutritionally secure in empowered households was 70% and 98% respectively. Conclusions: Developing programmes and policies to improve the range of domains of women’s empowerment requires a focussed policy agenda, bringing together policy makers from a number of different sectors including education, economy, communications, technology and agriculture. Women’s empowerment has the potential to make positive changes not only in food and nutrition security, but in all aspects of family health and wellbeing.

## Introduction

Over the last decade, food, and nutrition security (FNS) has gained prominence in policy and research and is considered an important pillar for health and well-being. The term FNS combines the features of both food security and nutrition security to emphasize that they are closely interlinked. ***Food security*** refers to the availability, access, utilization, and stability of food. ***Nutrition security*** refers towards adequate nutrient intake in terms of energy, protein, vitamins, and minerals for all people at all times.

Despite sufficient food production globally, the number of hungry people continue to rise and around 690 million people (8.9 percent) are suffering from hunger worldwide. Moreover, 2 billion of the population have no access towards safe and nutritious food [1]. Asia is the region reporting the highest share of world food insecurity [1]. Of the food insecure and undernourished countries of Asia, Pakistan is one of the most susceptible. It is ranked at 78 out of 113 countries and 88 out of 107 countries according to Global Food Security Index and Global Hunger Index [2, 3]. Moreover, the prevalence of undernourishment in Pakistan is 12.3% with 26.1 million undernourished people [1]. Despite being a self-sufficient country, 36.9% of the households are food insecure in Pakistan [4, 5]. Similarly, the number of children suffering from stunting and wasting are 9.5 million and 1.8 million, respectively [1]. Existing studies have documented that, when households with less access of affordable and nutritious food, they prioritise purchasing low cost staple foods (e.g. grains and rice) which are high in energy (calories) but low in micronutrients [6, 7]. This prioritization leads to micronutrient deficiencies, particularly in iron, iodine, zinc and vitamin A [6, 8, 9].

Women’s empowerment is a policy priority in many international organisations [10, 11]. Researchers have shown its strong relationship with food and nutritional security by highlighting the lack of opportunities for deprived and marginalised rural women compared to men [12]. Women’s empowerment has been demonstrated to reduce undernourishment [13, 14] and enhance food security [15, 16].

Rural women play a significant role in agriculture, representing 43% of agricultural labour force both globally and in developing countries [17] and, produce 50% of the world food [18, 19]. Rural women are involved in different types of agricultural activities and a variety of household tasks [20]. As women are not only food producers and care takers, but also an important determinant of household FNS, it is necessary to understand the role of rural women in Pakistan while assessing FNS at the household level. To do this, an understanding of the domains of female empowerment, and how to measure it is important.

There are several indices developed in the past to determine relevant dimensions of women’s empowerment in agriculture sector, some of them are: i) the “Women’s Empowerment in Agriculture Index (WEAI)”, ii) pro-WEAI which further developed WEAI to meet projects’ impact assessment needs, iii) Women’s Empowerment in Livestock Index (WELI) and iv) the Women’s Empowerment in Fisheries Index (WEFI). The WEAI was originally developed as a tool to monitor empowerment of rural women under the US government initiative called “Feed the Future” [21]. The degrees of men’s and women’s empowerment were then measured in several nations, including Bangladesh, Ghana, Uganda, Guatemala, and Pakistan, among others [13, 19, 20, 22, 23]. The WEAI consists of five dimensions that represent the empowerment status in agriculture sector only: (i) production (ii) resources (iii) income (iv) leadership, and (v) time use that represent the empowerment status in agriculture sector only.

However, we argue that a woman empowered in agriculture sector does not necessarily means that she is also empowered in other decision-making areas which are likely to impact on FNS.

In order to measure and capture multidimensional picture of women empowerment in rural sectors of Pakistan, we developed the “Rural Women Composite Empowerment Index (RWCEI)” which incorporates not only agriculture dimension, but also other dimensions such economic empowerment, autonomy, decision making, qualification, time allocation, mobility empowerment, political empowerment, awareness and violence.

There are three main gaps in the research related to female empowerment and FNS in rural areas. Firstly, previous studies have used proxy variables or WEAI to measure women’s empowerment but there is no research undertaken in Pakistan or elsewhere that has assessed women’s empowerment by considering dimensions which are more expansive than the rural focussed ones used in the WEAI for example. Secondly, whilst FNS has been analysed by various studies at the household level [4, 20, 24] limited research has been done in the context of female empowerment and evaluated the association between women’s empowerment and household FNS in rural Pakistan [20]. Thirdly, there is limited evidence of measuring the impact of communities on FNS by employing hierarchical structure of a data set which we will be doing as part of the research.

In order to measure and capture multidimensional picture of women empowerment in rural sectors of Pakistan, we developed the “Rural Women Composite Empowerment Index (RWCEI)” which incorporates not only agriculture dimension, but also other dimensions such economic empowerment, autonomy, decision making, qualifications, time allocation, mobility empowerment, political empowerment, awareness and violence.

The aim of the present study was to explore the relationship between women’s empowerment and food and nutrition security, in rural Pakistan.

The objectives were to use data from Pakistan Rural Household Panel Survey to:

1. develop and test (using factor analysis) a broader set of domains to create a Rural Women Composite Empowerment Index (RWCEI) to measure female empowerment in rural areas
2. Use dietary intake assessment together with number of food groups and recommended nutrient intakes to estimate household level FNS
3. To compare both food and nutrition security in regard to female empowerment (using the RWCEI)
4. To undertake a multi-level mixed effect regression models to explain the relationship between food security, nutrition security and women’s empowerment

## Methodology

### Data and study area

The data used in the analyses were taken from the Pakistan Rural Household Panel Survey (PRHPS) (IFPRI/IDS 2010–2014). The survey was conducted in rural areas of three provinces of Pakistan comprising of 19 districts: 12 districts of Punjab; two districts of Khyber Pakhtunkhwa (KPK); and five districts of Sindh. The Baluchistan province was not included in the sample due to security reasons. The survey was conducted in 76 Mouzas (A mouza is an administrative unit based on land revenue records and may correspond to a specific land with one or more settlements) with four mouzas randomly selected from each of 19 districts (S1 Table). The PRHPS used a multistage stratified technique for sampling. For this study we used data from third round of survey containing 1879 households. We compiled a data set by using women’s module, male modules, household level, and community level modules of the survey. The complete dataset is available in IFPRI dataverse archive and publicly accessible at: https://dataverse.harvard.edu/dataset.xhtml?persistentId=doi:10.7910/DVN/JWMCXY

PRHPS collected information on a variety of food items (that households were consumed) during the recall period of seven days listed in the household questionnaire. These household questionnaires included two further questionnaires i.e., male, and female questionnaires that are designed to collect individual and household level information from (main male and female) respondents separately. It contained 60 different types of foods and beverages considered as basic food items necessary for the dietary requirement of Pakistani people. In this part of the questionnaire, respondents were requested to think of all the food items and drinks that their family had consumed from their farm production, through market purchases, food gifts, and transfers. These food items were then converted into kcal according to the food consumption table of Pakistan [25]and analysed at the household level.

### Variable construction

In present study, we used food security and nutrition security as dependent variables and rural women composite empowerment index (RWCEI) as a key independent variable. How we constructed these variables is outlined in detail below.

#### Key independent variable

*Rural Women Composite Empowerment Index (RWCEI)*. In order to measure and capture a multidimensional picture of women empowerment in rural sectors of Pakistan, we developed the “Rural Women Composite Empowerment Index (RWCEI).” It is based on the WEAI developed by Alkire et al. and adapted by Nuzhat Ahmad & Huma Khan [21, 23] but includes a wider range of empowerment dimensions such economic empowerment, autonomy, decision making, qualification, time allocation, mobility empowerment, political empowerment, awareness and violence. The RWCEI therefore measures women’s empowerment through nine domains, fourteen sub-domains and eighty-nine indicators (S2 Table).

Many assessments of women’s empowerment have relied on a power typology founded in the seminal writings of Freire (1968) on autonomy and Lukes (1974) on power, and expressed with regard to gender and women’s empowerment by Rowlands (1995, 1997) [26–29]. This typology contrasts dominating or exerting "power over" others with creative types of empowerments such as "power within" (encompassing self-respect, identity, and a knowledge of rights), "power to" (implementing individual goals), and "power with" (acting collectively toward shared interests). Different studies also categorize types of empowerments in different dimensions of power [30–33].

In regard to power, empowerment can be framed in both positive and negative ways (as per Rowlands 1997). In a positive sense, agency occurs when people recognise their own worth and the purpose they bring to their acts (individual’s internal agency, or "power within") and are able to act to achieve their objectives (instrumental agency, or "power to"), even when others or social norms oppose them. In a negative sense, it refers to actors exceeding others’ agency and exerting control or "power over" their lives and resources [34]. Empowerment, then, is about changes in these various forms of power, which interact and reinforce one other to produce unequal outcomes.

With regard to the Rowlands (1997), Malapit et al. (2019), Meinzen-Dick et al. (2019), and Elias et al. (2021), theory of power, it is significant to substitute nine domains of RWCEI (in addition to those in WEAI) under the classifications of power [29–32]. The theory categorizes power (empowerment) into four dimensions, and every dimension is important while constructing the index. Rowlands (1997) states that empowerment is a power and power may be manifested in four ways: power within, power to, power with and power over. **Power within** relates to self-confidence and personal strength to make decisions. In this dimension, qualification and awareness domains are included. By getting an education and awareness a woman can gain self-confidence and can make better decisions in her life [30, 31, 33]. **Power to** (instrumental agency) means individual agency, and the capability to choose and carry out activities. So, in this category, domains of economic empowerment, autonomy, and decision-making lies. Moreover, the domain of time allocation and mobility empowerment also fits under this category. Time allocation domain is about woman having enough personal time for herself, and it is possible only if work is divided among family members [30–33]. Mobility empowerment means that women can move freely within and outside her settlement. It simply reflects the power to use one’s time for different activities and the power to go to different (important) places. Women’s capacity for decision making is enhanced if she is financially empowered and earning money. Moreover, autonomy is also important for decisions regarding spending money to buy her own personal things or not. Similarly, capability or capacity for performing actions is also reflected in whether she has power to make decisions about agricultural activities, household decisions and financial decisions [30–33].

**Power with** (power to participate in collective process) perceives that empowerment is a collective process that involves the support and interaction of families, societies, organizations, and communities. The domain of political empowerment fits under this category. Politically empowerment means that is she can vote and participate in political or other organisations and is not dependent on whether her family allows her to do so or not. **Power over** measures the degree of strength in relationships between woman and other members of family or community. Therefore, for this category violence domain is included. Violence refers to whether the woman is facing domestic violence in her family or not. If relationship with her family members is strong, then she is considered empowered in this category of power [30–33].

RWCEI was formulated in four steps. Firstly, we made binary responses of all indicators by assigning a value ‘1’ if woman had achievement/ adequacy in that indicator; otherwise ‘0’. The transformation of the variables to a binary format was necessary to create an “empowerment” variable. This was needed prior to the factor analysis.

Secondly, we summed all these binary scores under each sub-domain; a cut-off 30% was used to identify woman’s empowerment in each sub-domain. We made the judgement that if women had 30% adequacy in their responses, then she was considered empowered in that sub-domain. As there are no set standards/guidelines for deciding on such a threshold for a given index [35] we made this judgement based on a number of factors. Measurement or discussion of women’s empowerment often ignores the fact that women are tied together by strong emotional and structural bonds and their interests are strongly vested in their families which influences individual autonomy. Previous studies focus on individual empowerment irrespective of social status of the family but in Pakistan women rarely make any independent decisions. Joint family system is the bedrock of Pakistani societies [36]. Therefore, for devising the cut-offs for index we focused on norms of society, and as such a 30% cut-off level is more realistic. In absence of this support structure, the threshold may be higher. This does mean that our index has a relatively low threshold for women to be classified as ‘empowered’, but it is a starting point for measuring these important domains.

Thirdly, the same procedure was followed to identify woman empowered in each domain. Lastly, women’s empowerment index was calculated by using factor analysis with tetrachoric correlations. Weights were assigned to each domain based on their factor loadings and index was generated in STATA. A factor analysis of a matrix of tetrachoric correlations is more appropriate when input variables are dichotomous [37, 38]. Studies investigated that factor analysis is a useful technique for the construction of index [39].

#### Outcome variables

*a) Household food security*. To construct the household food security (*FS*_*h*_) variable, calorie intake per capita was calculated for each selected household in each region through the dietary intake assessment (DIA) approach. Quantities of food items were converted into grams from kg’s or litres and then into calories with the guidance of the food consumption table (FCT) for Pakistan [25]. FCT provides the calories amount of every food item in 100 grams of edible portion. Therefore, for each food item, calories per gram were computed. The data of food items was based on the 7-days recall period; therefore, household daily calorie intake was obtained by dividing the weekly data by seven and converted into a single unit i.e., kcal per day to obtain household daily calorie availability. Calculated calories were then converted into per adult equivalent (AE) by adjusting according to gender, age, and physical condition (pregnancy and lactation) of household members by using caloric adjustment table and expressed in adult equivalent units. Energy requirement depends on the sex and age of the person and therefore it varies across household members. To account for these changes adult equivalence scale was used in the development of this variable. Details about calculating calorie availability per adult equivalent (AE) are given in S1 Appendix.

Total calorie availability per day per adult equivalent *TC* (*tkcal*)_*h*_ at the household level “*h*” was measured by dividing the total calories of every household from the AE size of the respective household “*h*”.


$$TC{\left(tkcal\right)}_{h}=\frac{tkcal_{h}}{AE_{h}}$$ (1)

To estimate the household’s food insecurity status, *TC* (*tkcal*)_*h*_ was compared with minimum dietary energy requirement (MDER). MDER was the threshold level defined by FAO i.e., 1745 kcal per capita per day. It is the minimum amount of calorie requirement that is necessary to meet the adequate energy needs of a person. It was assumed that when the household was not capable enough to meet the MDER, it was unable to sustain its health and therefore it lied in the food insecurity group.


$$FS_{h}=TC{\left(tkcal\right)}_{h}-MDER$$ (2)

Where; *FS*_*h*_ is the food security status of *h*^*th*^ household, *TC* (*tkcal*)_*h*_ is total calorie intake of *h*^*th*^ household per day per adult equivalent and *MDER* is recommended threshold level of food security for the Pakistani population. A household unit was declared food secure if *FS*_*h*_ was greater than and equal to “0”. Furthermore, *FS*_*h*_ converted in to three categorical variables i.e., food insecurity, mild food security and high food security.

*b) Nutrition security*. Healthy food and good quality of diet are usually associated with a diversified diet and adequate amount of nutrient intake. Different indices were developed in the past to measure dietary diversity and quality e.g. Healthy Eating Index [40] and Chinese Diet Quality Index [41].

To construct the nutrition security variable, we created household dietary diversity scores (HDDS) and household dietary quality scores (HDQS). They serve as qualitative measures that exhibit household’s diversity among food items and food groups [42] and are simple tools that exhibit the dietary quality of households [43].

#### Household Dietary Diversity Score (HDDS)

Food consumption score (FCS) developed by the World Food Program (WFP) to measure the HDDS [44] was used in the present study. It measures the scores by assigning weights to food groups consumed by a household during the seven days. Eight food groups were deduced from twelve food groups, used by the present study on the grounds of nutritional attributes and proposed by FAO to measure the nutritional security at the household level [42]. There were standard weights designed for each food group that constituted the food consumption score and a continuous variable was generated with a possible range of 0 to 112. The food consumption groups include starches, pulses, vegetables, fruit, meat, dairy, fats, and sugar. The formula, based on these groups, with the standard weights, is:

$$FCS=\left(Starch\;Staples*2\right)+\left(Pulses*3\right)+\left(Vegetables*1\right)+\left(Fruits*1\right)+\left(\left(Meat/Fish\right)/Eggs*4\right)+Milk/Dairy*4)+\left(Fats*0.5\right)+\left(Sugar*0.5\right)$$ (3)

The reason for assigning unequal weights was that every food group did not have equal importance in calculating the total scores because of different nutritional attributes. For example, fats and sugar have less significance in a healthy diet as compared to milk, meat, and pulses. Therefore, accounting for these differences was crucial in estimating the true picture.

#### Household Dietary Quality Score (HDQS)

Household Dietary Quality Score (HDQS) is considered as an essential measure when assessing food and nutrition security. Different studies argue that people are consuming enough calories but still their diet is missing in essential nutrients. For example, if they are only taking cereals in their diets and do not include fruits and vegetables, milk and milk products, so their diet remains deficient in essential micro and macronutrients [6, 7]. Therefore, the required amount of nutrients are essential for a healthy and active human body. To measure whether a household is consuming the right amount of nutrients the study estimated macronutrients (proteins) and micronutrients including vitamins (A, B, and C) and minerals (calcium, iron, iodine, and zinc) in their diet plans, and utilised the Pakistan dietary guidelines with FAO standards for daily recommended allowances of these nutrients according to different age groups [45]. Each type of nutrient was converted into standard milligrams equivalents according to the food composition table (FCT) for Pakistan [25]. The daily intake of all household members was summed up and compared with the recommended daily nutrient intake (RNI) by age, sex, and physical condition (pregnancy and lactation) of household members.

Those households who fell below the recommended threshold level were considered nutrient-deficient households as they are consuming poor quality of diet. And those households who score above the threshold level were considered nutrient-rich households, as it was likely they were meeting their nutrient requirements and consuming good quality of diet.


$$HDQS_{h}\geq RNI_{h}=1$$ (4)

$$HDQS_{h}<RNI_{h}=0$$ (5)

*HDQS* is the score of households ‘*h*’, and it equals one when its score is equal to or greater than recommended nutrient Intake (*RNI*_*h*_). After creating the nine macro and micronutrient variables, we then developed a household dietary quality index through principal component analysis (PCA) and used this index as a dependent variable in the analysis [46]. PCA is used as it provides a better approximation of the required factors [47]. It uses Pearson correlations for identifying important factors that contains most of the information [48–50]. It is also most appropriate technique for the construction of indices [39]. Different studies also used PCA for construction of FNS indices in history [50, 51].

#### Modelling

*Multi-level mixed-effect logistic regression methodology*. Different control variables were used in the analysis that are crucial to incorporate according to previous studies. For example, household size and its occupation [4, 52–55], unemployment to employment ratio [56], employment status of household head [57], expenditures on daily food items [58], household wealth index [21, 59], educated and health communities [58, 60, 61] etc. Description and measurement of these control variables are given in S3 Table. As described previously, we had three dependent variables in the analysis and the type (numerical, categorical etc.) of all variables was different. We used ordered logistic, linear, and binary logistic regression for FS, HDDS, and HDQS respectively. To incorporate the hierarchical composition of the data set (i.e. individuals were nested in households and households were nested in the 19 communities) multi-level modelling was adopted as suggested by Kamanda, Madise, and Schnepf [60]. For this purpose, we added the role of health facilities as a proxy for identifying food insecure households in the community and role of educated women in the community. A community with educated women was included, because a higher level of women education is associated with better health outcomes and wellbeing of their families [57, 60].

Three models were used for comparison. **Model 1** is the food security (FS) model and it focused on calorie intake by the households. **Model 2** is the dietary diversity model (HDDS) and it focused on a number of food groups consumed by the households. **Model 3** referred to the dietary quality (HDQS) and it described nutrient intake by the households.

The two-level model is written as follows:

$$logit\;\left(\pi_{ik}\right)=\text{log}\left[\frac{\pi_{ik}}{1-\;\pi_{ik}}\right]=\beta_{0}+\beta_{i}RWCEI_{i}+X_{ik}+\mu_{0k}$$ (6)

Where *π*_*ik*_ is the probability of household FNS for the *i*_*th*_ household in the *k*_*th*_ community. *RWCEI* is the key independent variable and *X*_*ik*_ is the vector of covariates corresponding to the *i*_*th*_ household in the *k*_*th*_ community, *β*_*o*_ is the vector of parameters and *μ*_*ok*_ is the random effect at the community level. The intercept (average probability) of being food and nutrition secure was assumed to vary randomly across communities. The fixed effects (measures of association) were expressed in odds ratios (OR) and 95% confidence interval (95% CI). The random effects (measures of variation) are expressed as variance coefficients. We used the post estimation command to determine the p-value of variance.

## Results and discussion

### Descriptive statistics

**Fig 1:**
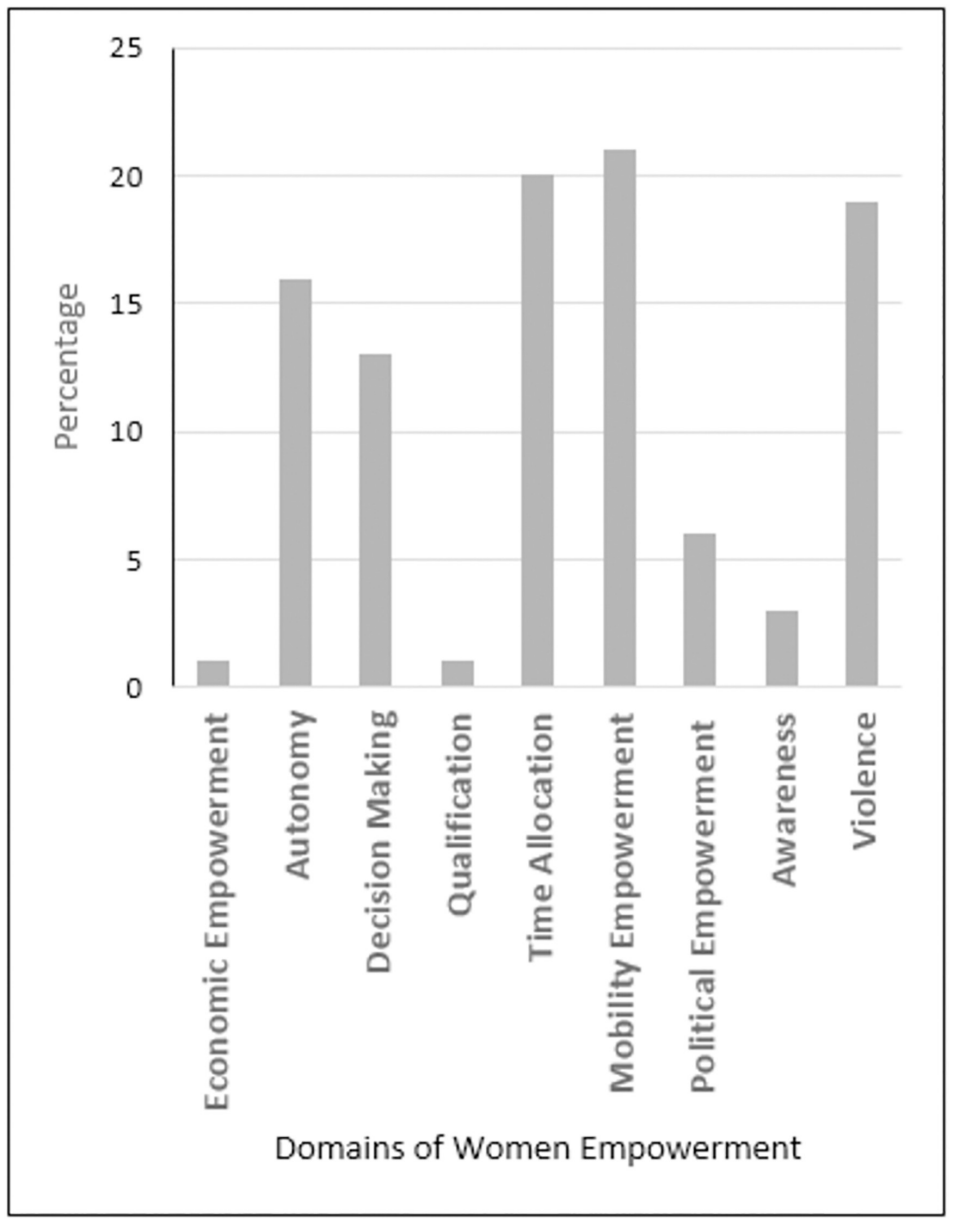
Proportion of domains explaining Rural Women’s Composite Empowerment Index. Source: Authors own calculations.

As mentioned earlier, women’s empowerment was the key independent variable in the analysis and to estimate its relationship with household FNS we develop an index called Rural Women Composite Empowerment Index (RWCEI) by using factor analysis. Factor loadings and weights of all domains are given in S4 Table. Results show that that mobility empowerment (21%), time allocation (20%), and violence (19%) domains contribute the most to women empowerment in rural Pakistan. Violence domain operates in the inverse direction, indicating the disempowerment level of a woman. Therefore, in order to make a direct relationship with women empowerment we included women who *didn’t* face domestic violence in this category. We observed that, taken together, they are 60% empowered in these domains (Fig 1).

Descriptive statistics are presented in Table 1. The results show that 54% of the sampled households are involved in agricultural occupations; while 85% of household’s heads are employed and earning an income; and 25% of households are in the rich category of the wealth quantile. In our study, around 44% communities have a proportion of women with secondary or higher education, and 44% communities have basic health facilities for their members. Furthermore, Table 2 highlights that only 33% of households are experiencing high food security. Maximum food consumption scores are 107 out of 112 and minimum are 5.5. Moreover, 50% prevalence of undernourishment is observed in terms of macro and micronutrients in the sampled households. Our results indicate that vitamin A is rich in their diets but there was minimum intake of iodine (1%).

**Table 1:** Summary statistics of the sampled households (n = 1879) and communities (n = 19).

Variables	Mean	Std. Dev.	Min	Max	Freq.	Percentage
**Household Level Variables**						
Women Empowerment	1.683	0.351	0.055	1.999		
Family Size	7.257	3.322	2	37		
Household Occupation (Agriculture)	0.500	0.500	0	1	1,020	54.23
Unemployment to employment ratio	3.331	2.304	0	11		
HH Head employment status (Employed)	0.852	0.355	0	1	1,603	85.22
Household food expenditures	5.560	0.552	1.168	8.462		
Wealth Index						
Poorest	0.250	0.433	0	1	471	25.04
Poorer	0.247	0.432	0	1	465	24.72
Middle	0.252	0.434	0	1	474	25.20
Rich	0.251	0.434	0	1	473	25.15
**Food Security**						
Food Insecurity	0.308	0.462	0	1	627	33.33
Mild Food Security	0.308	0.462	0	1	627	33.33
High Food Security	0.308	0.462	0	1	627	33.33
Calorie supply (kcal/day/AE)	1843.234	1209.316	428.0024	42178.51		
**Nutrition Security**						
Food Consumption scores	67.01	14.08	5.5	107		
Prevalence of undernutrition	0.505	0.500	0	1	931	49.49
*Macro and Micronutrients*						
Protein intake (mg/day/AE)	0.682	0.466	0	1	1,280	68.23
Calcium intake (mg/day/AE)	0.211	0.408	0	1	395	21.06
Iron intake (mg/day/AE)	0.779	0.415	0	1	1,461	77.88
Iodine intake (mg/day/AE)	0.011	0.103	0	1	20	1.07
Zinc intake (mg/day/AE)	0.094	0.292	0	1	176	9.38
Vitamin A intake (μg RE/day/AE)	0.954	0.209	0	1	1,790	95.42
Vitamin B intake (mg/day/AE)	0.700	0.458	0	1	1,314	70.04
Vitamin C intake (mg/day/AE)	0.366	0.482	0	1	687	36.62
**Community level variables**						
Educated Community	0.438	0.496	0	1	825	43.86
Healthy Community	0.485	0.499	0	1	831	44.18

**Table 2:** Results from multi-level mixed-effect models of food security and nutrition security are depicted.

	Food Security		Nutrition Security		
	*Caloric Intake (Model 1)*		*HDDS (Model 2)*	*HDQS (Model 3)*	
Predictors	Coefficient (SE)	Odd Ratio (95% CI)	Coefficient (SE)	Coefficient (SE)	Odd Ratio (95% CI)
**Fixed Effect**					
**Household Predictors (N = 1879)**					
RWCEI	0.529** (0.255)	1.698** (1.030–2.799)	2.481* (1.432)	0.684** (0.299)	1.981** (1.103–3.559)
Family Size	-0.408*** (0.022)	0.665*** (0.636–0.695	-0.167* (0.100)	-0.444*** (0.028)	0.641*** (0.607–0.678)
Household Occupation Agriculture)	0.462*** (0.116)	1.587*** (1.264–1.992)	0.028 (0.673)	0.513*** (0.137)	1.669*** (1.277–2.184)
Unemployment to employment ratio	-0.036* (0.022)	0.965* (0.924–1.007)	-0.235** (0.127)	-0.098*** (0.026)	0.906*** (0.861–0.954)
Head employment status	0.448*** (0.141)	1.565*** (1.187–2.063)	1.392* (0.836)	0.404** (0.169)	1.497** (1.076–2.084)
Household food expenditures	2.241*** (0.136)	9.406*** (7.201–12.285)	7.859*** (0.658)	1.547*** (0.151)	4.696*** (3.493–6.314
Wealth Index					
Poorest	1	1	1	1	1
Poorer	0.097 (0.148)	1.102 (0.825–1.471)	1.289 (0.874)	0.121 (0.174)	1.129 (0.802–1.588)
Middle	0.084 (0.156)	1.087 (0.802–1.475)	4.033*** (0.927)	0.153 (0.184)	1.166 (0.812–1.674)
Rich	0.336** (0.165)	1.399** (1.013–1.934)	6.569*** (0.985)	0.596*** (0.196)	1.814*** (1.234–2.667)
Region of residence					
Punjab	0.963 (0.807)	2.618 (0.538–12.747)	1.168 (3.082)	0.444 (0.839)	1.559 (0.301–8.064)
KPK	1	1	1	1	1
Sindh	1.406 (0.926)	4.079 (0.664–25.065)	3.861 (3.906)	1.195 (1.059)	3.303 (0.414–26.355)
**Community Predictors (N = 19)**					
Educated Community	0.909** (0.439)	2.481** (1.048–5.878)	3.031* (1.714)	1.385** (0.596)	3.994** (1.242–12.843)
Healthy Community	0.579** (0.292)	1.785** (1.006–3.166)	2.832** (1.405)	1.121*** (0.426)	3.069*** (1.333–7.067)
**Random Effect**					
**Community Level**					
Variance (SE)	1.174* (0.634)		27.445*** (11.033)	2.059* (1.131)	
VPC (%)	20.05		15.75	17.44	
**Model Fit Statistics**					
AIC	3412.635		**14794.18**	**2045.773**	
Wald chi2	429.75		**329.19**	**300.31**	
(Prob > chi2)	0.0000		**0.0000**	**0.0000**	
Log-Likelihood	-1690.318		-7381.088	**-1007.887**	

**Note:** Intercept cut points are excluded from the output

For Model ‘1’, ‘2’ and ‘3’ we used ordered, linear and binary logistic regressions respectively.

Abbreviations: SE = Standard Error, CI = Confidence Interval, VPC = Variance Partition Coefficient, AIC = Akaike Information Criteria.

* = p <0.1,

** = p <0.05,

*** = p <0.01

**Fig 2:**
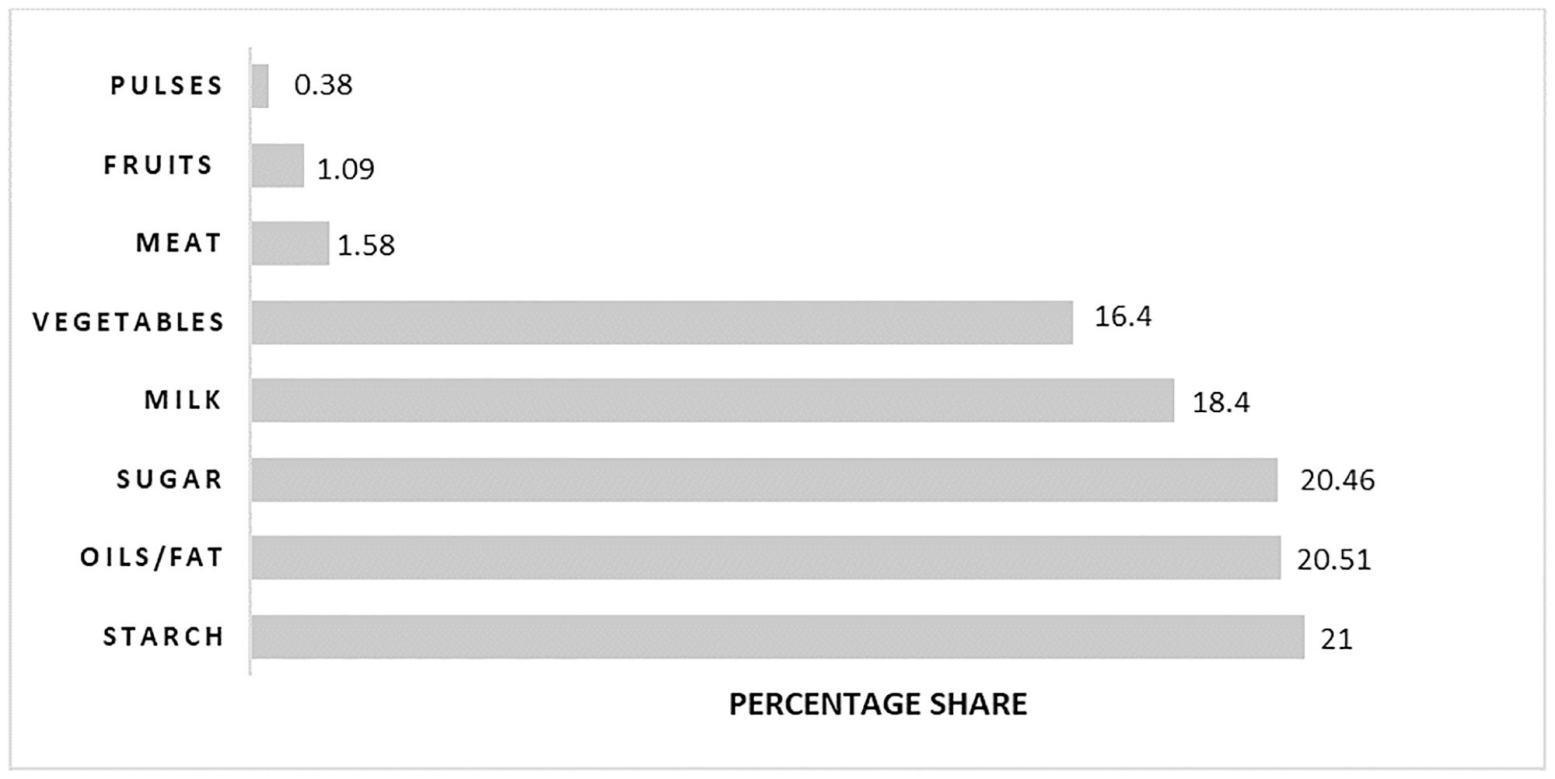
Percentage share of eight food groups in the diets of rural households. Source: Authors own calculations.

The share of each group in the diets are reported in Fig 2, which shows that major share in the diets of rural people was from sugar, fats/oils and starch, and their diets are deficient from healthy food items like fruits, pulses, and meat. Because of their low income and limited access to affordable nutritious food, families placed emphasis on purchasing low-cost food items that are rich in starch but low in micronutrients. This in turn results in various micronutrient deficiencies, especially of iodine, iron, and zinc [6, 61–63].

### Household FNS and women’s empowerment

We adopted the multi-level mixed effect regression models to explain the relationship between food security, nutrition security and women’s empowerment. Such type of models are useful when data is nested within groups [64]. In our case, households were nested in 19 communities a two-level multilevel logistic regression was employed with households at level 1 and communities at level 2. Results for food security are presented by using caloric intake as response variable and nutrition security are presented by using dietary diversity and dietary quality as response variables in model 1, 2 and 3 respectfully. The fixed effects (measures of association) are expressed both in coefficients and odds ratio (OR) with their respective standard errors (SE) and 95% confidence intervals (95% CI) in parenthesis. The random effects (measures of variation) are expressed as Variance Partition Coefficient (VPC). The odd ratio explains the probability of an outcome to happen with respect to independent variables. It gives the percentage change in probability when an explanatory variable increases by one unit and is a useful method to interpret the results [64]. Table 2 shows the results from all three models. The Data file and STATA do file are attached in supporting information for reference (S1 and S2 Data).

We used three different multi-level models depending on the nature of dependent variables. As food security variable has three categories (1 = food insecurity, 2 = mild food security and 3 = high food security), we used ordered logistic regression. For representing nutrition security, we had two variables i.e., household dietary diversity scores (HDDS) and household dietary quality scores (HDQS). For HDDS we used linear regression because this dependent variable is in continuous form, and we adopted binary logistic regression for HDQS because of dichotomous nature of this variable. The total variance(τ) in food and nutrition security associated with the community level was initially calculated by using the null model for all dependent variables which includes no variables and focus only on decomposing the total variance in community components. The variance for all models was significant across the communities i.e., Model 1: τ = 0.379 ρ = 0.01, Model 2: τ = 26.296 ρ = 0.001, Model 3: τ = .365 ρ = 0.01 and variance partition coefficient (VPC) or intra-community correlation explains that 9.7%, 13.2% and 9.9% variation in FNS is explained by variation in communities respectively. It means that variation in FNS is occurring due to the community levels, indicating that differences in communities are explaining the likelihood of improving FNS.

Table 2 presents results for three models of FNS by employing RWCEI as key independent variable, household level characteristics along with community level covariates and their slopes were allowed to vary to explore their effects across communities. Our results demonstrate that women’s empowerment (RWCEI) increases the likelihood of high food security and nutrition security as the variable is statistically significant and had a positive relationship in all models. Different studies considered women’s empowerment as an important determinant of FNS at household level and they revealed that it is positively linked with food availability in terms of calories and nutrients, therefore, women empowerment has a direct link in improving household FNS [22]. Our results augment this argument by showing that women’s empowerment is associated with an increase in caloric intake, higher dietary diversity, and quality. Empowered women had an increased probability of 70% for maintaining household food security, it means that empowered women can play a pivotal role through her authorities in increasing household caloric intake. Similarly, by increasing one unit of RWCEI, dietary diversity increases by 2.48 units and there is 98% probability that household’s dietary quality is guaranteed with women’s empowerment.

Employment status of household head is a significant variable in defining the status of household FNS. It ensures that household had enough income to maintain the consumption patterns, moreover, income growth through employment is important for the household nutrition both in terms of quantity (calories) and quality (nutrients) [65]. Our results support this argument by indicating that employed household head had 57% higher probability of explaining higher level of food security than unemployed head, and with each additional member of family, probability of moving towards high food security decreases by 34%. The same result is also reported in previous study, that as family size increases, food security deceases [56]. Similarly, there are 50% chances of achieving dietary quality through nutrient intake if household head is employed and addition of new member in a family is associated with 36% lower probability of dietary quality. Same is the case with HDDS as the scores increases by 1.392 units if household head is employed and decreases by 0.16 units for a new additional member in a family.

Agricultural occupation of a household had a positive impact on FNS. The hypothesis is that households involved in agricultural farming have more opportunities to get diversified food because they can grow different types of fruits and vegetables in their own farms instead of purchasing from market with money. Our results demonstrate that with agricultural farming as an occupation, households had 59% of higher probability of achieving high food security as compared to the non-agricultural livelihoods. Similarly, the probability of nutrient intake increases by 67% with agricultural occupation. As agricultural occupation had no impact on dietary diversity of households, potentially because it does not ensure the daily consumption of adequate food groups. However, if a household has earning members and have enough income, then it can spend money on purchasing food items to meet dietary requirements of its family members. This hypothesis is captured by household’s expenditures on food items in the model. Previous studies investigated that, households involving in agricultural occupation have good food choices and more food expenditures, moreover, with income from agricultural activities they start investing on quality of food, and preference about healthy nutritious food increases as compared to less healthy food [20, 22]. Our findings are aligned with these studies and show positive and significant relationship between expenditures on health, food items and caloric intake, dietary diversity, and dietary quality [66]. With each unit increase in expenditures, the probability of high food security and dietary quality increases by 9.4 and 4.7 odds respectively and dietary diversity by 7.85 units.

Dependency on earning members by non-earning members of the family is captured by unemployment to employment ratio in the model and results show that it has a significant negative impact on the household FNS. With an increase of more non-earning members, ratio increases and with unit increase in ratio, the probability of getting high food security decreases by 4%, nutrition security by 0.235 units and 10%, through dietary diversity and quality respectively. Similarly, household wealth had a positive and significant impact on FNS. Our results show that fourth category (i.e., rich) of wealth quantile is significant only in all models, while first three quantiles are insignificant. Our results are in-line with study [58], that at lower wealth levels only basic necessities are fulfilled and as wealth increases people start spending on health and lifestyles. So, in our case results show that only rich households had 40% higher probability of high food security, 6.56 units increase in dietary diversity and 81% more likelihood of dietary quality.

To analyse the impact of contextual variation on household FNS, we finally introduced “Level 2” in the model, which incorporated community-level variables i.e., proportion of women who are educated (secondary or higher) and the number of health facilities in a community. Households belong to the community in which the proportion of educated women was higher had a higher likelihood i.e., 150% of getting high food security as compared to those communities in which there was no secondary or higher female education. Similarly, educated community increases the dietary diversity and quality in household’s diets. The hypothesis is that an educated community influences positively on women’s empowerment which in turn affects the knowledge about adopting the nutrient rich consumption patterns and healthy diets for family. This result is aligned with study [59, 60, 67] which state that with an increase in the proportion of mothers’ education in the community, maternal and children’s health seeking behaviour improves. Moreover, Sinharoy et al. in a study [39] argued that women’s schooling was positively and significantly associated with individual and household diets. Household’s well-being, healthy diets and better lifestyle are also associated with health facilities in a community. Because these facilities can provide awareness about quality diets and helps households to seek better health practitioner at the time of need. It reduces the prevalence of food insecurity from the community and helps in promoting healthier environment [68]. Therefore, it is necessary to have as many as facilities as possible in a community. Our results indicate that if a community has at least 15 small health facilities in the form of private pharmacy/drugstore, doctor, lady health worker, private clinic, trained midwife, public dispensary, basic health unit, traditional healer, traditional birth attendant etc. then there is 79% higher probability of getting high food security as well as dietary diversity increases by 2.832 units and 200% more chances household’s dietary quality.

For the sake of robustness, we adopted a model discrimination approach which involved comparing the model diagnostics with, and without, the community variables. For this purpose, we dropped our community variables from all models to check that either our models are better fit without contextual variations or not. We compared AIC values between original and reduced models, and we find that original models performed better with contextual variations as AIC values are lower in these models (S5 Table).

## Strengths and limitations of the study

The present study adds to the available research in four different ways. First, this is one of the few studies that emphasises the multidimensional nature of women empowerment. Second, the present study analyses the role of rural women in improving household FNS. Third, we incorporated the hierarchal structure of the data set by employing multilevel regression models. It is assumed that households are nested with in the communities, and it is necessary to incorporate the impact of communities on household FNS. Lastly, we have used dietary intake assessment together with number of food groups and recommended nutrient intakes to estimate household level FNS after adjusting adult equivalent factors. This is the unique as it compares both food and nutrition security at a time. According to the best of our knowledge no single study has been conducted previously in Pakistan evaluating the association of a multidimensional women’s empowerment index with household FNS in a community framework.

Our study has some limitations, each of which opens avenues for future research. First, the results constructed here are based on PRHPS data collected in 2014. The purpose of utilizing this data is that it is rich on the topics on women’s empowerment and household FNS variables. This unique type of data has not been updated yet. However, there are other surveys which includes variables on these topics, but they are not rich enough to contain and cover all domains (especially in case of women empowerment). Further studies can use the latest data if it is updated to include questions which facilitate policy makers in understand the relation between women’s empowerment and FNS. Secondly, the sampled data is limited to three provinces of rural Pakistan, and therefore research at larger scale (including rural areas of all provinces) is needed to generalize the findings. Thirdly, the choice of 24-hour vs 7 days’ recall period can be utilized in future studies to compare the results. Moreover, Adult Equivalent scale and food consumption table need to be revised according to latest population energy requirements. Last our decisions as to what threshold met the definition of empowerment may have been too high or too low and may need adjusting in future studies.

## Conclusions and policy implications

From the past many years, Food and Nutritional Security and women’s empowerment have been at the forefront of international and national policy agendas. FNS remains a prominent goal in the Millennium Development Goals (MDGs) as well as in the Sustainable Development Goals (SDGs). Despite long-held interest in increasing household FNS, there is a limited understanding of the factors affecting it. Women’s empowerment is found to be one crucial factor in determining whether a household is FNS or not. We have illustrated that it is crucial to understand the complex associations between FNS and the multiple domains of women’s empowerment. Our paper adds important insights into the domains of women’s empowerment that are associated with FNS. The rural women composite empowerment index (RWCEI) can guide policy makers to have a nuanced understanding of the domains in which policy is needed, and it can also be used to measure the progress. For example, the study indicated that rural women are disempowered in education, mass media awareness and credit access (S4 Table), therefore policies should be drafted accordingly. The present study proposed some key recommendations to strengthen the status of rural women in Pakistan. First, provision of paid jobs and access to credit should be emphasized while designing policies. Second, educational opportunities in terms of different programs and vocational trainings need to be introduced. Last, there is a need of time to reshape the community’s thinking about women’s empowerment. It can be possible through public awareness campaigns.

There is an important policy message in this research: empowering of women in rural areas can result in households that are more food and nutritionally secure. Through women’s empowerment, the probability of increasing nutrition security in terms of nutrient consumption is more (98%) relative to the food security in terms of caloric intake (70%). Therefore, policies should be designed in such a way that keep focus on improving nutrition of families rather than focus on just increasing calories.

Developing programmes and policies to improve domains of women’s empowerment requires a focussed policy agenda, bringing together policy makers from a number of different sectors including education, economy, communications, technology and agriculture. Women’s empowerment is the key to making positive changes not only in FNS but in all aspects of health and wellbeing.

## Supporting information

S1 Appendix Calorie availability per adult equivalent. (DOCX)

S1 Table List of districts included in the survey. (DOCX)

S2 Table Weights of domains and variables of rural women composite empowerment index (RWCEI). (DOCX)

S3 Table Description and measurement of control variables used in the analysis. (DOCX)

S4 Table Factor loadings of RWCEI domains. (DOCX)

S5 Table Reduced models of Food and Nutrition Security (FNS) by using OLS. (DOCX)

S1 Data Data file (Stata). (DTA)

S2 Data Do file (Stata). (DO)
