# Supplementary material for: The relationship between women’s empowerment and household food and nutrition security in Pakistan
Source: PLoS One. 2022 Oct 20;17(10):e0275713. doi: 10.1371/journal.pone.0275713 (PMC9584378; doi:10.1371/journal.pone.0275713)
Supplement: S1 Appendix — (DOCX) [file pone.0275713.s001.docx]

**Calorie availability per Adult Equivalent (AE)**

Energy requirement depends on the sex and age of the person and therefore it varies across household members. To account for these changes adult equivalence scale was used in the development of this variable. The quantity of calorie availability i.e., kcal/day of each household expressed in kcal/day for each adult equivalent by dividing daily household calorie availability by adult equivalent units. This method is useful because it measures actual food consumption by every household member along with their dietary quality. The adjustments of per capita units according to the adult equivalence scale were important otherwise it would underestimate the actual per person caloric requirement by overlooking the difference caused by household composition [48].

Adult Equivalent size of household was calculated by using the following equation:

$${AE}_{h}=\sum_{i=1}^{hhsize} {AE}_{i} (1)$$

Adult equivalent number (${AE}_{i}$) for each household member was calculated from the calorie requirement scale for each age group mentioned in the Pakistan dietary guideline issued by the collaboration of the government of Pakistan and FAO [41] (S1 Appendix Table). Adult equivalent size/factor compares every person’s energy needs from each household with that of an adult male with moderate activity i.e., 2251 kcal per day in 2014. Adult equivalent size (${AE}_{h})$ was calculated by summation of adult equivalence numbers of individuals at household level. It allowed DIA comparisons among households by controlling sex-age differences.

**Table:** Adult equivalent scale to calculate daily calorie requirements with respect to age and gender

| Age (Years) | Body Weight (Kg) | Calories*(kcal) | AE  Conversion Factors |
| --- | --- | --- | --- |
| Children | | | |
| 0-6 months | 6.0 | 524 | 0.2327 |
| 6-11 months | 8.9 | 708 | 0.3145 |
| 1-3 | 12.1 | 1022 | 0.4540 |
| 4-6 | 18.2 | 1352 | 0.6006 |
| 7-9 | 25.2 | 1698 | 0.7543 |
| Boys | | | |
| 10-17 | 49.7 | 2824 | 1.2545 |
| Girls | | | |
| 10-17 | 46.7 | 2326 | 1.0333 |
| Men | | | |
| 18-59 | 65 | 3091 | 1.3731 |
| 60 and over | 65 | 2496 | 1.1088 |
| Women | | | |
| 18-59 | 55 | 2408 | 1.0697 |
| 60 and over | 55 | 2142 | 0.9515 |
| Pregnant (+278) * | | | |
| 12-17 | - | 2604 | 1.1568 |
| 18-59 | - | 2686 | 1.1932 |
| 60 and over | - | 2420 | 1.0750 |
| Breastfeeding (+450) ** | | | |
| 12-17 | - | 2776 | 1.2332 |
| 18-59 | - | 2858 | 1.2696 |
| 60 and over | - | 2592 | 1.1514 |
| National Average*** |  | **2251** | 1.0000 |

Source: Pakistan Dietary Guidelines for Better Nutrition by FAO and GOP (2018)

*Additional 278 kcal is required for pregnant women according to Pakistan Dietary Guidelines for Better Nutrition and it is with the collaboration of FAO and Government of Pakistan.

**Additional 450 kcal is required for lactating women according to Pakistan Dietary Guidelines for Better Nutrition and it is with the collaboration of FAO and Government of Pakistan.

***According to FAO, Average dietary energy requirement for Pakistan is 2243 in 2017. <http://www.fao.org/economic/ess/ess-fs/ess-fadata/en/#.XSgRiOgzbIU>

There are different threshold levels designed for estimating the dietary intake, used by various researchers at different times. For the present study, the benchmark given by Food and Agriculture Organization (FAO) for Pakistan i.e., 2251 Kcal/capita/day was used as a normative reference for sufficient nutrition, called average dietary energy requirement (ADER). (FAO data file for ADER and MDER: <http://www.fao.org/economic/ess/ess-fs/ess-fadata/en/#.YCvWh2j7TIU>)

Calorie requirement per capita was divided by ADER to obtain adult equivalent factors for every age group. Then multiplied by the number of household individuals in each age category by corresponding AE factors and sum the number of AE to obtain the total number of AE for each household.
