# Supplementary material for: The relationship between women’s empowerment and household food and nutrition security in Pakistan
Source: PLoS One. 2022 Oct 20;17(10):e0275713. doi: 10.1371/journal.pone.0275713 (PMC9584378; doi:10.1371/journal.pone.0275713)
Supplement: S1 Table — (DOCX) [file pone.0275713.s002.docx]

| District Names | Households | District Names | Households | District Names | Households |
| --- | --- | --- | --- | --- | --- |
| Punjab |  | Sindh |  | Khyber Pakhtunkhwa (KPK) |  |
| Attock | 112 | Dadu | 112 | Mansehra | 112 |
| Bahawalnagar | 111 | Hyderabad | 112 | Nowshera | 112 |
| Bhakkar | 112 | Jacobabad | 110 | **Total KPK** | **224** |
| Dera Ghazi Khan | 108 | Sanghar | 111 |  |  |
| Faisalabad | 102 | Thatta | 112 |  |  |
| Jhang | 111 | **Total Sindh** | **557** |  |  |
| Kasur | 106 |  |  |  |  |
| Khanewal | 106 |  |  |  |  |
| Multan | 111 |  |  |  |  |
| Rahim Yar Khan | 107 |  |  |  |  |
| Sargodha | 111 |  |  |  |  |
| Vehari | 112 |  |  |  |  |
| Total Punjab | **1309** |  |  |  |  |
