# Supplementary material for: The relationship between women’s empowerment and household food and nutrition security in Pakistan
Source: PLoS One. 2022 Oct 20;17(10):e0275713. doi: 10.1371/journal.pone.0275713 (PMC9584378; doi:10.1371/journal.pone.0275713)
Supplement: S2 Table — (DOCX) [file pone.0275713.s003.docx]

| Domains | Sub-Domains | Variables (coding: yes = 1; no = 0) | Weights |
| --- | --- | --- | --- |
| Power to | | | |
| Economic Empowerment  weight = $\frac{\mathbf{1}}{\mathbf{22}}$  Variables= 17 | **Access**  **Ownership** | **Women access to paid farm work (No. of days per annum (Rabi & Kharif 2012-2013)** Sowing  Weeding  Harvesting  Post-Harvest Activities  Other Activities  **Women Access to paid non-farm work**  No. of days/annum  **Woman access to own business (handicraft, weaving, cottage activities**  Ownership (Yes/No)  Type of Ownership (Sole Proprietor, Jointly with a household member, Jointly with an outsider)  **Ownership of Valuable Assets**  Agricultural land  Large livestock (oxen, buffalo, etc.)  Small livestock (goat, sheep, etc.)  Farm equipment  House  Consumer durables  **Saving**  **Bank Account**  **Bisi*** | **1/374**  **1/374**  **1/374**  **1/374**  **1/374**  **1/374**  **1/374**  **1/374**  **1/374**  **1/374**  **1/374**  **1/374**  **1/374**  **1/374**  **1/374**  **1/374**  **1/374** |
| Autonomy  Weight = $\frac{\mathbf{1}}{\mathbf{13}}$  Variables =7 | **Authority**  **Decision Making** | **A woman can spend money to buy:**  Food from market  Clothes for herself  Medication for herself  Toiletries/ cosmetics for herself  **Would your husband or in-laws allow you to engage in a business or earn additional money from working inside your home?**  **Involvement in a job that brings in cash or in-kind income?**  **The decision about the earned income?** | **1/91**  **1/91**  **1/91**  **1/91**  **1/91**  **1/91**  **1/91** |
| Decision Making  Weight= $\frac{\mathbf{1}}{\mathbf{23}}$  Variables= 24 | **Agriculture Decision Making**  **Financial Decision Making**  **Household Decision Making** | **Woman participation in decisions about:**  Food crop farming  Cash crop farming  What inputs to buy for agriculture production  When or who would take crops to the market  Livestock raising  **The extent to which woman can influence decisions regarding:**  Food crop farming  Cash crop farming  What inputs to buy for agriculture production  When or who would take crops to the market  Livestock raising  **Women decision making about:**  Non-farm business activities  Wage/Salary  Borrowing money or item  What to do with the borrowed money or item  **Woman decision making about:**  Food for household  Clothing for household  Household occasional small expenditures  Household occasional large expenditures  Renovation or maintenance of the house  Girls marriage  Female children education  Male children education  Household health care or medication  Method of contraception to be used | **1/552**  **1/552**  **1/552**  **1/552**  **1/552**  **1/552**  **1/552**  **1/552**  **1/552**  **1/552**  **1/552**  **1/552**  **1/552**  **1/552**  **1/552**  **1/552**  **1/552**  **1/552**  **1/552**  **1/552**  **1/552**  **1/552**  **1/552**  **1/552** |
| Time Allocation  Weight = $\frac{\mathbf{1}}{\mathbf{20}}$  Variables = 3 |  | Time spends on household chores  Time spends on household agricultural activities  Time spends on non-agricultural activities | **1/60**  **1/60**  **1/60** |
| Mobility Empowerment  Weight = $\frac{\mathbf{1}}{\mathbf{2}}$  Variables =11 |  | Does a woman feel safe walking/traveling alone within your settlement?  Does a woman feel safe walking/traveling alone outside your settlement?  How safe do women feel when visiting the following places? (neighborhood market, a neighboring village, field/farmland (own) and (landlord), sell products at the local mandi, traveling to the main city, participation in religious events, hospitals, ceremonies, attend the meeting | **1/22**  **1/22**  **(1/22 *9)** |
| Power Within | | | |
| Qualification  Weight = $\frac{\mathbf{1}}{\mathbf{71}}$  Variables = 5 | **Skill**  **Education** | **Ability to:**  Read  Write  To do basic calculations  Highest class attended  Technical or Vocational skill | **1/355**  **1/355**  **1/355**  **1/355**  **1/355** |
| Awareness  Weight = $\frac{\mathbf{1}}{\mathbf{19}}$  Variables=4 | **Community**  **Mass Media** | Are you aware of the presence of any community organization in your area?  Do you listen radio, watch TV or read newspaper? | **1/76**  **(1/76 *3)** |
| Power With | | | |
| Political Empowerment  Weight = $\frac{\mathbf{1}}{\mathbf{20}}$  Variables = 6 | **Participation**  **Perception** | Political Participation (yes/no)  Did women vote in the previous general elections (2008)?  Did the opinions of family members influence woman vote decisions?  To what extent do women feel her involvement in community groups and her influence in decisions affecting all villagers?  How satisfied is a woman with the community group's efforts to help her community during this 12-month period?  Do women think membership of any community group has changed her position in the household in any way? | **1/120**  **1/120**  **1/120**  **1/120**  **1/120**  **1/120** |
| Power Over | | | |
| Violence  Weight = $\frac{\mathbf{1}}{\mathbf{10}}$  Variables = 12 | **Domestic Violence (by husband)**  **Wife Beating**  **Domestic Violence (by other family members)** | Has your husband done something to humiliate you in front of others?  Has your Husband threatened to hurt or harm you or someone you care about?  Has your husband insulted you or made you feel bad about yourself?  Has your husband ever push you, shake you, or throw something at you?  Has your husband ever slap you?  Has your husband ever twist your arm or pull your hair?  Has your husband punch you with his fist or with something that could hurt you?  Has your husband ever kick you, drag you, or beat you up?  Has your husband try to choke you or burn you on purpose?  Has your husband ever threaten or attack you with a knife, gun, or other weapon?  Has your husband ever force you to have sexual intercourse or perform any other sexual acts with him when you did not want to?  Did anyone else inside your household ever do any of the above-mentioned things to hurt you? | **1/120**  **1/120**  **1/120**  **1/120**  **1/120**  **1/120**  **1/120**  **1/120**  **1/120**  **1/120**  **1/120**  **1/120** |

***** A form of group savings where individuals contribute collectively and receive a lump sum in turns.
