# Supplementary material for: The relationship between women’s empowerment and household food and nutrition security in Pakistan
Source: PLoS One. 2022 Oct 20;17(10):e0275713. doi: 10.1371/journal.pone.0275713 (PMC9584378; doi:10.1371/journal.pone.0275713)
Supplement: S3 Table — (DOCX) [file pone.0275713.s004.docx]

| Variables | Description | Measurement | Previous Studies |
| --- | --- | --- | --- |
| RWCEI | Rural women composite empowerment index | Index (0.055 - 1.999)  Continuous variable | [49], [62] |
| Household Size | Number of household members in a family | Continuous variable | [48], [49], [62], [50], [51] |
| Occupation of HH | Household either involved in agricultural occupation or not | Agricultural occupation =1; otherwise =0 | [4], [50], [51], |
| Unemployment to employment ratio | Ratio of non-earning to earning members of a household | Continuous variable | [52] |
| HH Employment Status | Head of HH involve in primary or secondary occupation (Agriculture, non-agriculture, Private, business entrepreneurship) | Yes=1; No=1 | [53] |
| HH Food Expenditures | Expenditures on daily food items | Continuous variable | [54] |
| HH Wealth Index | Information about livestock assets, house ownership and quality of house material, sanitary condition, quality of water and energy source | 4 quantiles generated by using PCA | [21], [55] |
| Educated Community | Proportion of women with secondary or higher education in a community | A community in which at least 5% of women are educated (secondary or above) is considered as educated community  Yes=1; No=1 | [56], [53] |
| Healthy Community | Number of health facilities available in a community (private pharmacy/drugstore, doctor, lady health worker, private clinic, trained midwife, public dispensary, basic health unit, traditional healer, traditional birth attendant etc.) | A community in which above 15 health facilities were present was considered healthy community  Yes=1; No=1 | [61] |
