# Supplementary material for: The relationship between women’s empowerment and household food and nutrition security in Pakistan
Source: PLoS One. 2022 Oct 20;17(10):e0275713. doi: 10.1371/journal.pone.0275713 (PMC9584378; doi:10.1371/journal.pone.0275713)
Supplement: S4 Table — (DOCX) [file pone.0275713.s005.docx]

| Domains | Factor Loading | Weights |
| --- | --- | --- |
| Economic Empowerment | 0.1470 | 0.0464 |
| Autonomy | 0.2570 | 0.0811 |
| Decision Making | 0.1394 | 0.0440 |
| Qualification | 0.0445 | 0.0140 |
| Time Allocation | 0.1751 | 0.0552 |
| Mobility | 1.7599 | 0.5557 |
| Political Empowerment | 0.1589 | 0.0502 |
| Awareness | 0.1660 | 0.0524 |
| Violence | 0.3191 | 0.1007 |
| Sum  Eigen Value  Variance %  Kaiser-Meyer-Olkin (KMO) adequacy  Bartlett’s Sphericity Test:  Approx. Chi-Square  Degree of freedom  Significance | 3.1669  3.391  61.47  0.5  1814.556  36  0.000 | 1.0000 |

KMO value for the sample is 0.5 i.e. equal to the threshold level and meets the required criteria for factor analysis. Bartlett's test of Sphericity in this study shows a significant value of chi-square (1814.56) at a significant p-value (0.000), which suggests that the population correlation matrix is not an identity matrix. Both tests show there is sample adequacy for factor analysis and strengthens the appropriateness of the use of factor analysis in this study.
