# Supplementary material for: The relationship between women’s empowerment and household food and nutrition security in Pakistan
Source: PLoS One. 2022 Oct 20;17(10):e0275713. doi: 10.1371/journal.pone.0275713 (PMC9584378; doi:10.1371/journal.pone.0275713)
Supplement: S5 Table — (DOCX) [file pone.0275713.s006.docx]

|  | **Food Security** |  | **Nutrition Security** | |
| --- | --- | --- | --- | --- |
|  | *Caloric Intake (Model 1a)* |  | *HDDS (Model 2a)* | *HDQS (Model 3a)* |
| Predictors | **Coefficient (SE)** |  | **Coefficient (SE)** | **Coefficient (SE)** |
| RWCEI | 0.517 (0.218) ^***^ |  | 0.149 (1.396) | 0.655 (0.253) ^***^ |
| Family Size | -0.369 (0.021) ^***^ |  | -0.226 (0.103) ^**^ | -0.407 (0.026) ^***^ |
| Household Occupation  Agriculture=1 | 0.612 (0.094) ^***^ |  | -1.334 (0.606) ^**^ | 0.739 (0.109) ^***^ |
| Unemployment to employment ratio | -0.028 (0.021) |  | -0.178 (0.133) | -0.092 (0.024) ^***^ |
| Head employment status  Yes=1 | 0.602 (0.132) ^***^ |  | 1.712 (0.861) ^**^ | 0.565 (0.154) ^***^ |
| Household food expenditures | 2.130 (0.116) ^***^ |  | 8.943 (0.615) ^***^ | 1.574 (0.129) ^***^ |
| Wealth Index  Poorest  Poorer  Middle  Rich | 1  0.006 (0.129)  -0.187 (0.130)  -0.089 (0.133) |  | 1  0.803 (0.856)  2.968 (0.863) ^***^  5.449 (0.882) ^***^ | 1  -0.0003 (0.150)  -0.198 (0.151)  0.029 (0.155) |
| Model Fit Statistics |  |  |  |  |
| AIC | 3522.922 |  | 14972.17 | 2134.678 |

*= p <0.1, ** = p <0.05, *** = p <0.0**1**
